# Supplementary material for: Revealing the molecular interplay of coverage, wettability, and capacitive response at the Pt(111)-water solution interface under bias
Source: Commun Chem. 2025 Feb 24;8:58. doi: 10.1038/s42004-025-01446-w (PMC11850831; doi:10.1038/s42004-025-01446-w)
Supplement: Supplementary file 2 — Supplemental Material [file 42004_2025_1446_MOESM2_ESM.pdf]

Revealing the Molecular Interplay of Coverage, Wettability, and Capacitive Response at the Pt(111)-Water Solution Interface under Bias

Federico Raffone,[1] Remi Khatib,[2,†], Marialore Sulpizi,[3] and Clotilde S. Cucinotta[1,*]

[1]Department of Chemistry and Thomas Young Centre, Imperial College London, White City Campus, London W12 0BZ, UK

[2]Department of Physics, Johannes Gutenberg University, Staudingerweg 9,

[†] Present address: 4 rue Roland Oudot 94000 Créteil France

[3]Department of Physics, Ruhr-University Bochum, Bochum Universitätsstr. 150, Germany

[*] E-mail: c.cucinotta@imperial.ac.uk

While electrified interfaces are crucial for electrocatalysis and corrosion, their molecular morphology remains largely unknown. Through highly realistic ab-initio molecular dynamics simulations of the Pt(111)-water solution interface in reducing conditions, we reveal a deep interconnection among electrode coverage, wettability, capacitive response, and catalytic activity. We identify computationally the experimentally hypothesised states for adsorbed hydrogen on Pt, H_{UPD} and H_{OPD}, revealing their role in governing interfacial water reorientation and hydrogen evolution. The transition between these two H coverage regimes with increasing potential, induces a shift from a hydrophobic to a hydrophilic interface and correlates with a change in the primary electrode screening mechanism. This results in a slope change in differential capacitance, marking the onset of the experimentally observed peak around the potential of zero charge. Our work produces crucial insights for advancing electrocatalytic energy conversion, developing deep understanding of electrified interfaces.

Supplementary Information

Supplementary Figures, Discussions and Tables

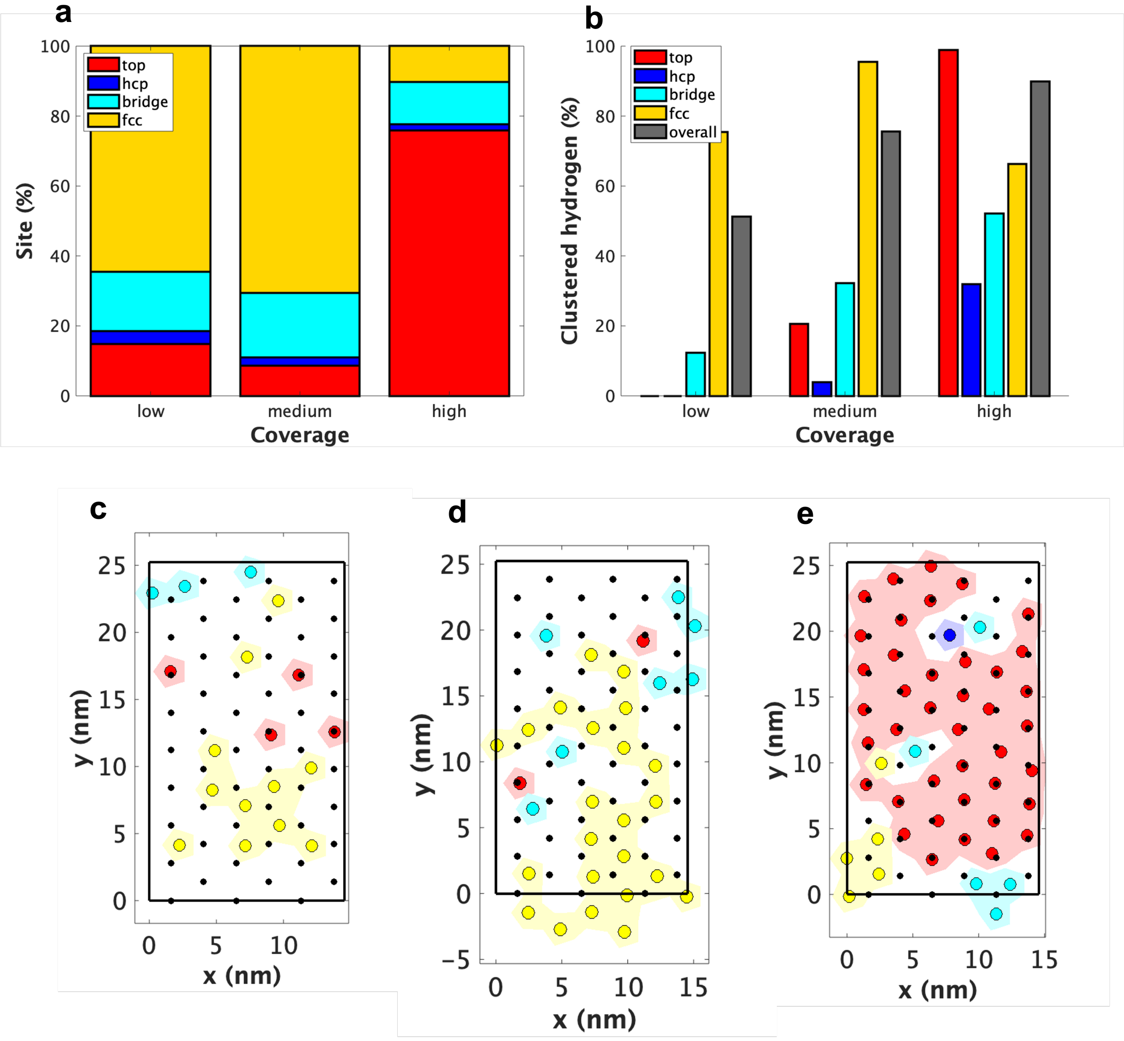

Figure S1:
Distribution of adsorbed Hydrogen atoms on the Pt surface versus coverage. (a). Percentage of hydrogen atoms belonging to a cluster (b). Example of low (absolute coverage 0.33 ML) (c), medium (absolute coverage 0.67 ML) (d) and high (absolute coverage 0.9 ML) (d) coverage surface with adsorbed atoms. The shaded area identify a cluster. The small black dots indicate the Pt surface atoms while the larger circles refer to the adsorbed hydrogen. The color code corresponds to the sites indicated in panel a and b.

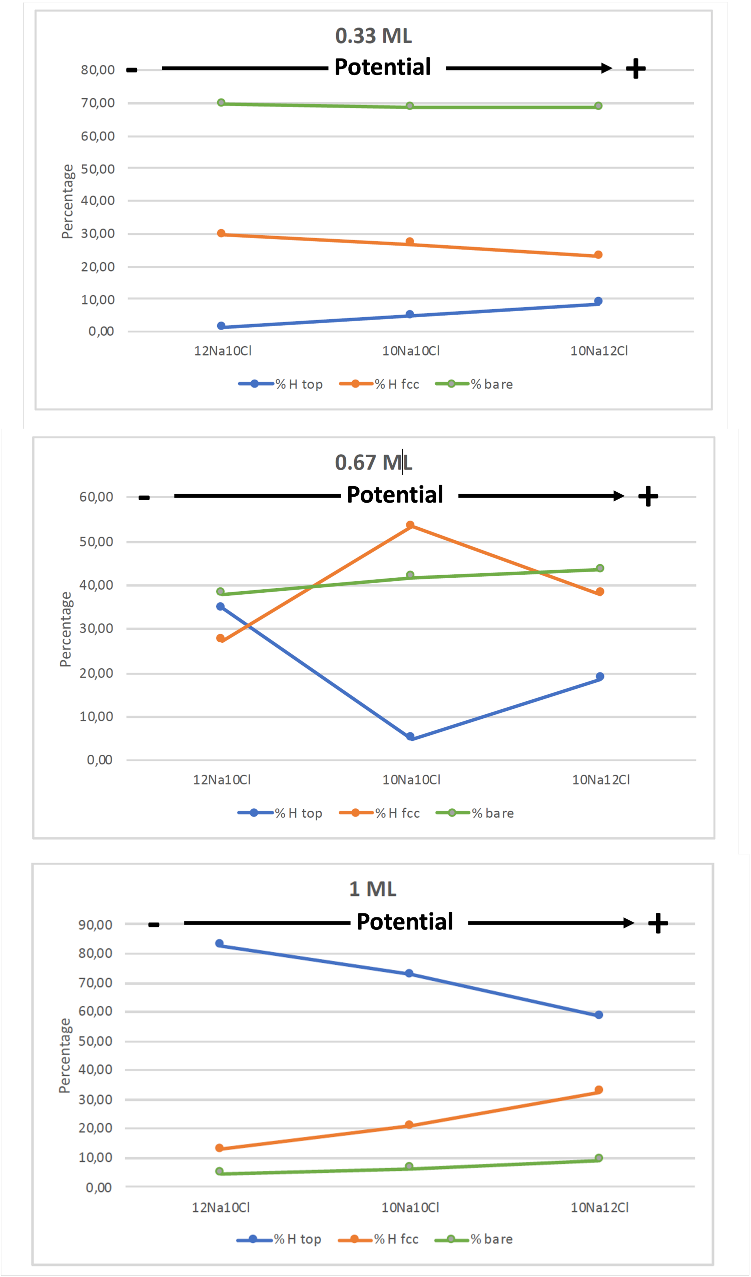

Figure S2: **Effect of varying ionic imbalance on Hydrogen site occupation.** Impact of varying ionic imbalance in the solution (10Na:12Cl, 10Na:10Cl, 12Na:10Cl) on the relative occupancy of fcc (H_{UPD}) and atop (H_{OPD}) hydrogen sites. Each inset illustrates H redistribution at the given total H coverage as the potential changes (by varying the ion imbalance in solution). The specific coverage indicated in the inset title.

Supplementary Discussion 1: Effect of local voltage variations on the distribution of H_{UPD} and H_{OPD} sites at low, medium and high H coverage.

To analyse how small voltage deviations from each coverage's equilibrium value influence the distribution of H_{UPD} and H_{OPD} sites, short AIMD simulations lasting approximately 5 ps (after equilibration) were performed. The primary dataset, analysed in the main paper, was developed using a concentration of 10Na:10Cl. Additionally, for each hydrogen coverage, variations in ionic concentrations of 10Na:12Cl and 12Na:10Cl were studied to generate potentials slightly lower and higher than that associated with the 10Na:10Cl condition, as per Khatib et al. (*?*).

It is worth noting, that although the concentration of Na and Cl ion is varied similarly for the each H coverage, the amount of H dissolved in solution in each H coverage is different, therefore the three H coverages correspond to different potential ranges, as described in Figure 4 and in the method section in the main text.

Figure **??** represents the impact of such potential variation on the on the percentage of fcc (H_{UPD}) and atop (H_{OPD}) sites in H chemisorption. We observe that for each coverage, the size of the predominant cluster increases when the potential decreases and the 0.66 ML coverage represents the boundary between the two regimes. In particular, at 0.33 ML coverage and high potentials (top part of the figure), it was observed that an increase in potential led to a decrease in the size of the H_{UPD} cluster compared to the H_{OPD} cluster. At 0.67 ML coverage and intermediate potentials (middle part of the figure), an interesting inversion in trend was noticed specifically at the 10Na:10Cl ionic concentration. Below this point, the size of H_{UPD} cluster increased while the H_{OPD} cluster decreased with increasing potential. Conversely, above this potential the trend reversed, showing a decrease in H_{UPD} cluster size and an increase in the H_{OPD} cluster with potential increase. At a coverage of about 1 ML (0.9 ML) and low potentials (bottom part of the figure), a decrease in potential resulted in a larger of H_{OPD} cluster and and a smaller H_{UPD} cluster.

The simulations revealed a dynamic interplay between potential and the distribution of fcc and atop sites during hydrogen chemisorption at different coverages.

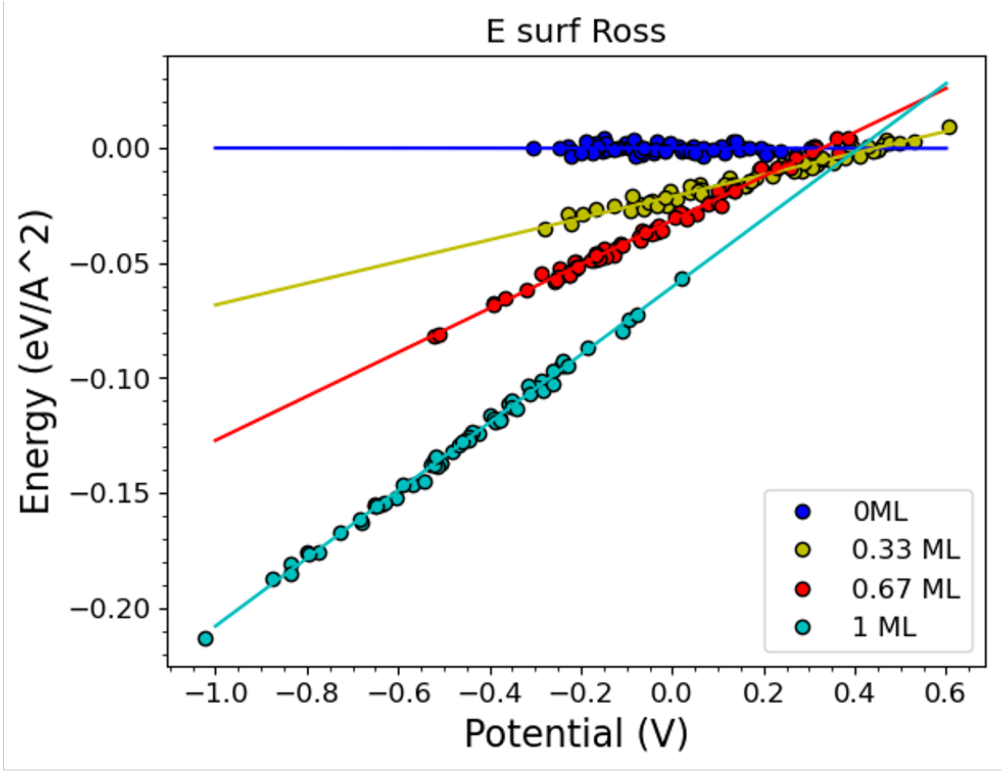

Figure S3: **Variation of surface formation energy with voltage**. The surface formation energy for H adsorption. Circular markers represent snapshots from the trajectory. In this graph the 0 of the potential scale is aligned with the average potential for the 0 coverage surface and the surface work function correction has not been applied yet, so the data points result shifted with respect to Figure 2 in the main paper. A linear interpolation of the dataset is depicted by a solid line.

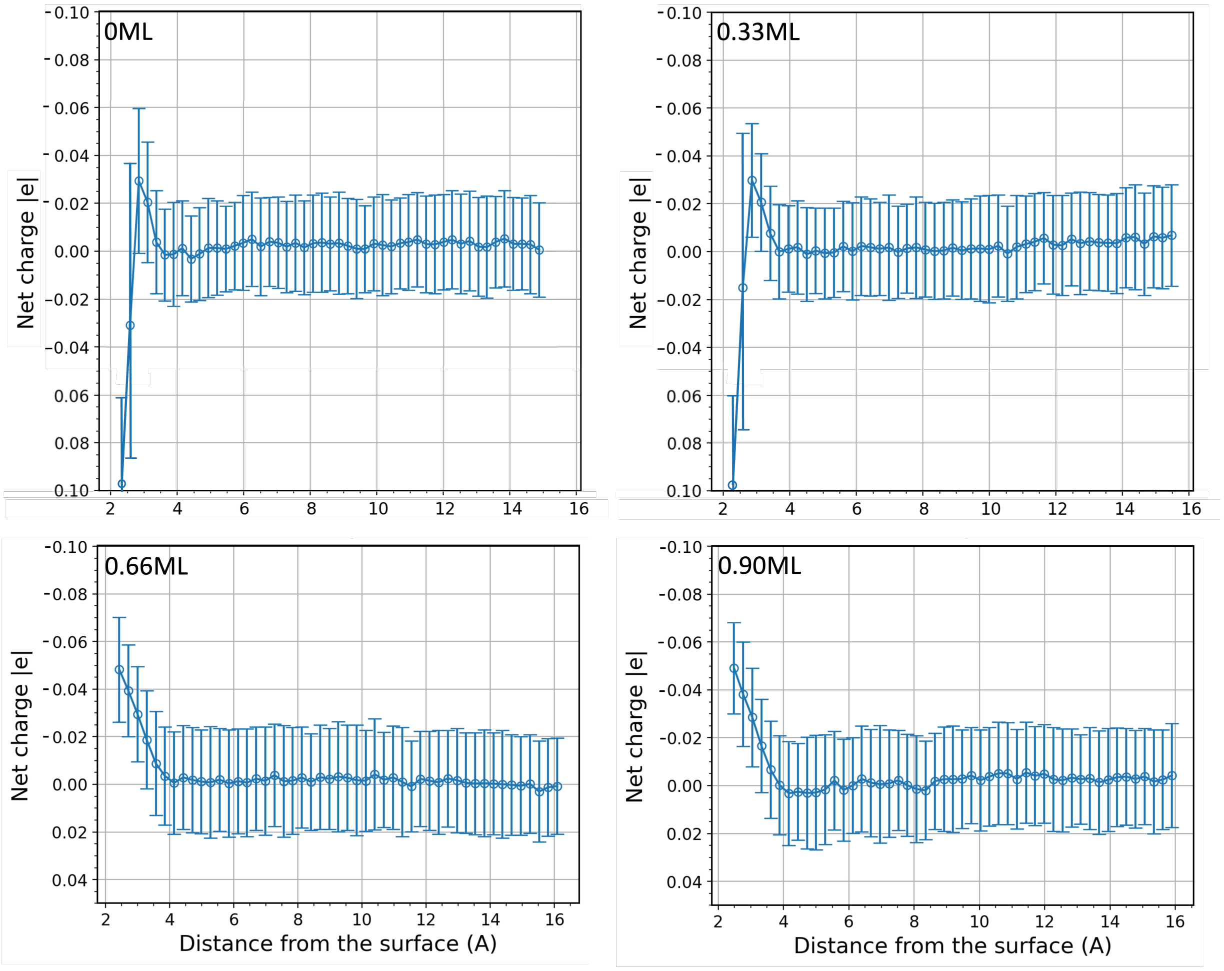

Figure S4: **Average charge distribution of the water molecules in the direction z perpendicular to the metal surface.** The net charge over water molecules in units of electrons, $|e|$, is represented as a function of the distance from the metal surface. The H coverage of each considered configuration is specified in each inset.

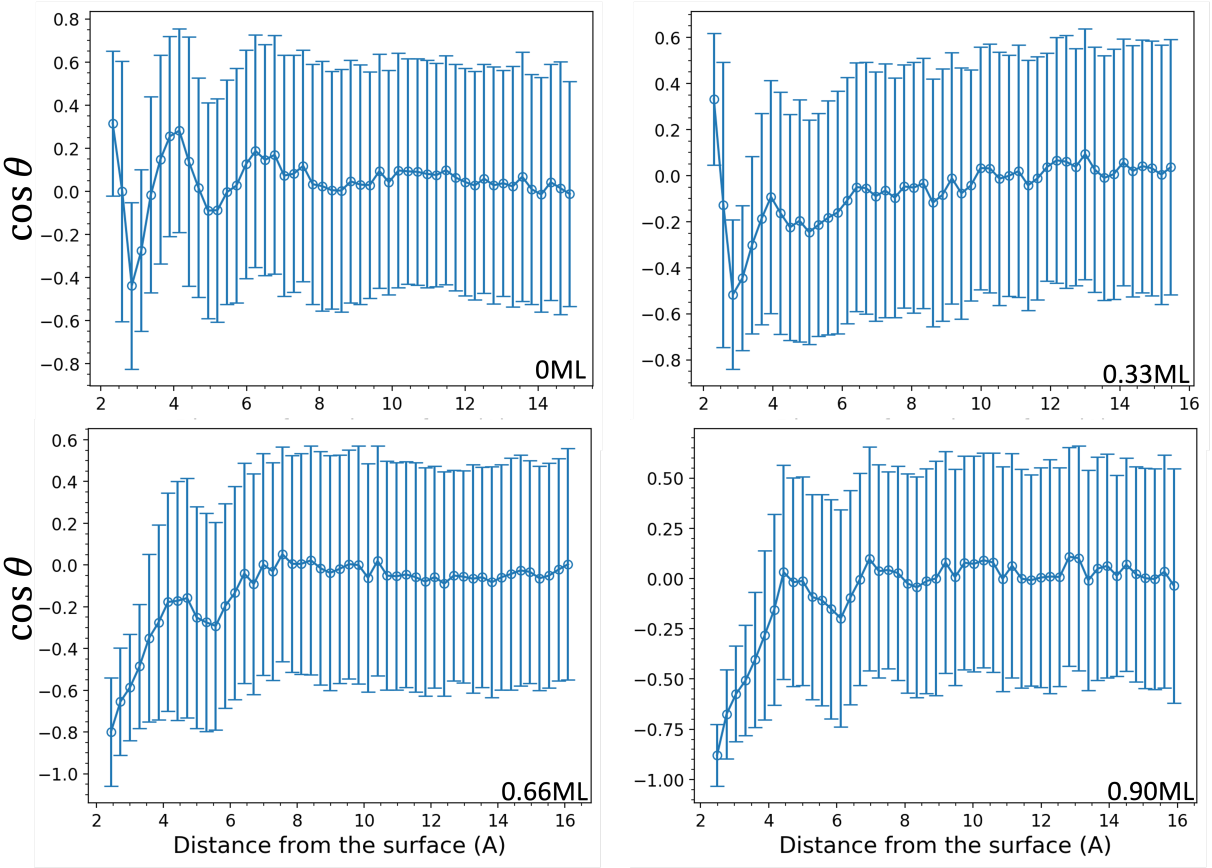

Figure S5: **Average angular distribution of the water molecules in the direction z perpendicular to the metal surface.** The H coverage is specified in each inset. This figure shows that there are two water adsorption regimes on top of Pt(111)/H surface: At low potentials which correspond to up to 0.66 ML H coverage, water is approximately found at 3 Å from the surface, and is only slightly negatively charged. For higher potentials, i.e. at coverages of 0.33 and below, water gets closer to the surface (to approximately 2.2 Å) and becomes negatively charged.

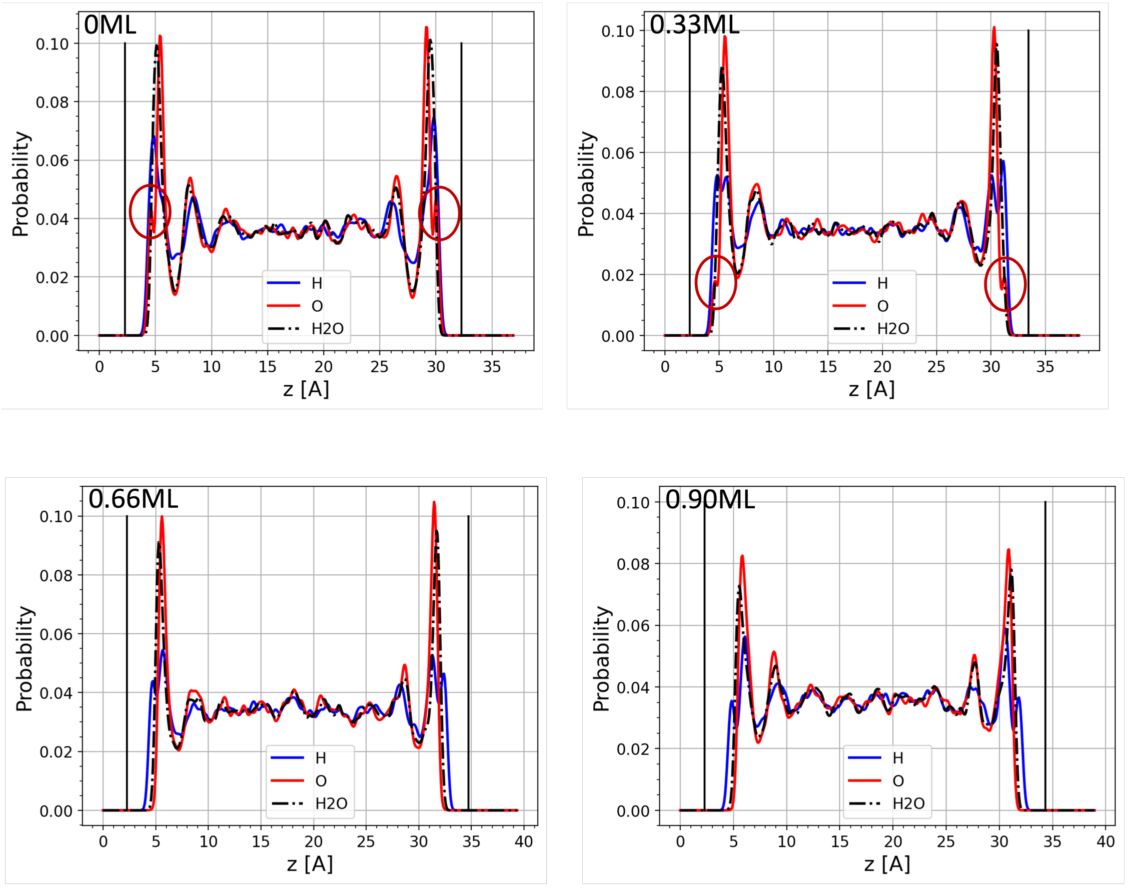

Figure S6: **Average mass distribution of the water molecules as a function of distance from the metal surface.** The blue, red and black dashed-dotted lines represents H, O and H2O distributions, respectively. The black vertical line signals the position of the Pt surface. The H coverage is specified in each inset. This mass distribution shows that there are two water adsorption regimes on top of Pt(111)/H surface. The red circles depicted in the O ML and 0.33 ML configurations (upper part of Figure **??**), highlight the position of the Oxygen atoms in the first chemisorbed water layer at approximately 2 Å from the Pt surface, as described under Figure S5. Notably the peaks are higher for 0 ML, confirming that the number of chemi-sorbed water molecules increases with potential. Looking at the alignment of the first H (blue) and O peaks (red), it can be seen that water in this first water layer lays almost flat. The first water peak is absent in the 0.66 ML and 0.90 ML configurations, and the position second water layer is signaled by an O peak at approximately 3 Å from the metal surface. The alignment between the first H and O peak, shows that the H atoms in this water layer point towards the metal surface (first blue peak from the metal surface) or lay in the same plane of the water layer (second blue peak from the metal surface).

Supplementary discussion 2: H coverage and surface hydrophilicity

The charge, angle and mass distribution described in Figures **??**, **??** and **??**, support the idea that the transition between the two H coverage regimes corresponds to a modulation in the hydrophilic character of the surface. Notably, water charging distribution is very similar at 0 and 0.33 ML coverage. Please note that the presence of a charge dot with large variance at approximately 2.5 Å from the surface is an artifact of the data representation, and does not correspond to any real water molecule.

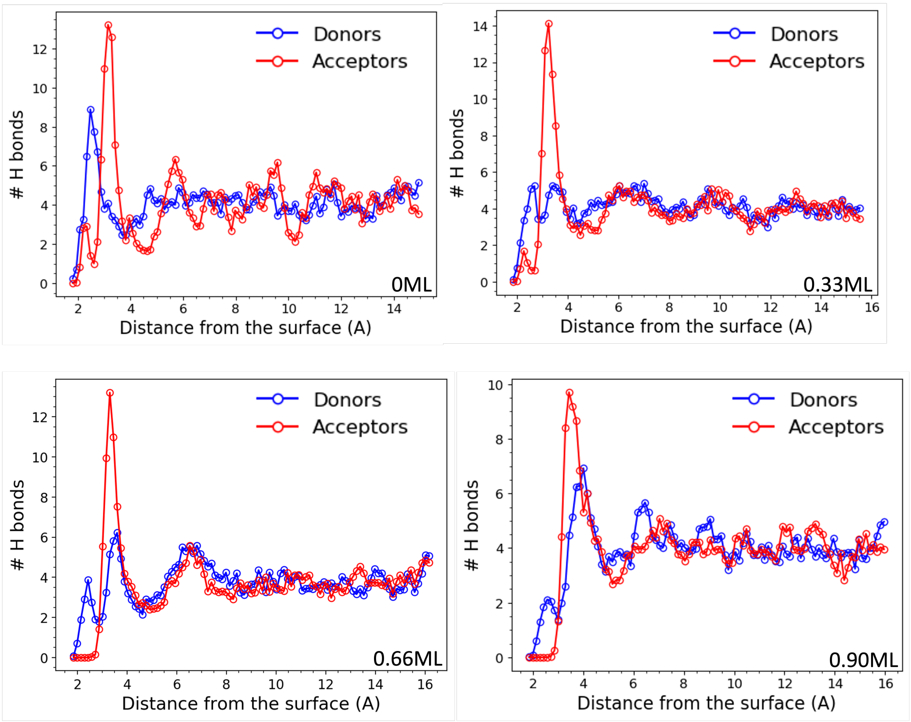

Figure S7: **Distribution of H donors and acceptors.** Average number of H bonds donors (red) and acceptors (blue) as a function of the distance from the metal surface. The H coverage is specified in each inset.

The figure shows that the number of available acceptors in the first water layer decreases from 0 ML to 0.33 ML configurations and goes to zero at higher coverages. This is consistent with having a decreasing number of water molecules pointing H outward from the surface and chemisorbing through the O atom, as H coverage increases (or potential decreases).The count of of H donors, progressively decreases with increasing H coverage (or decreasing potential). This corresponds to a decreasing number of H available to form H bonds with other water molecules when the first water layer is missing. Note that this figure restricts the count of H bonds only to the water molecules of the liquid and do not include possible H bonds with the surface. See also Supplementary Figure **??**.

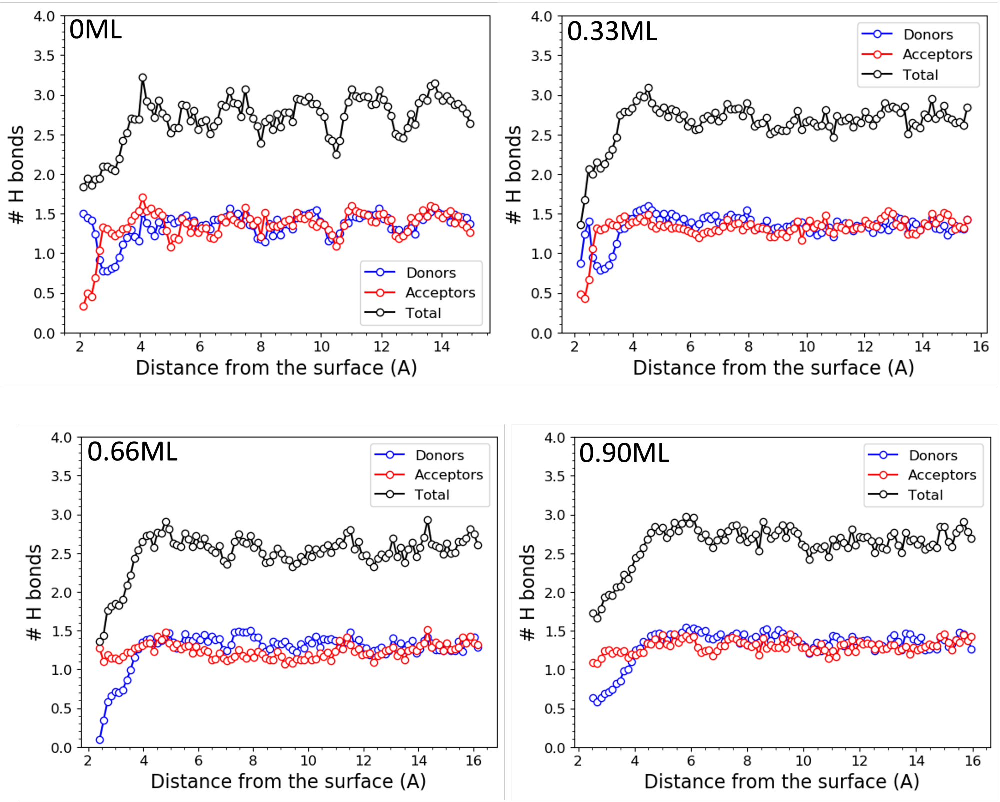

Figure S8: **Distribution of H donors and acceptors per molecule.** Average number of H bonds donors (red) and acceptors (blue) per molecule as a function of distance from the metal surface. The H coverage is specified in each inset.

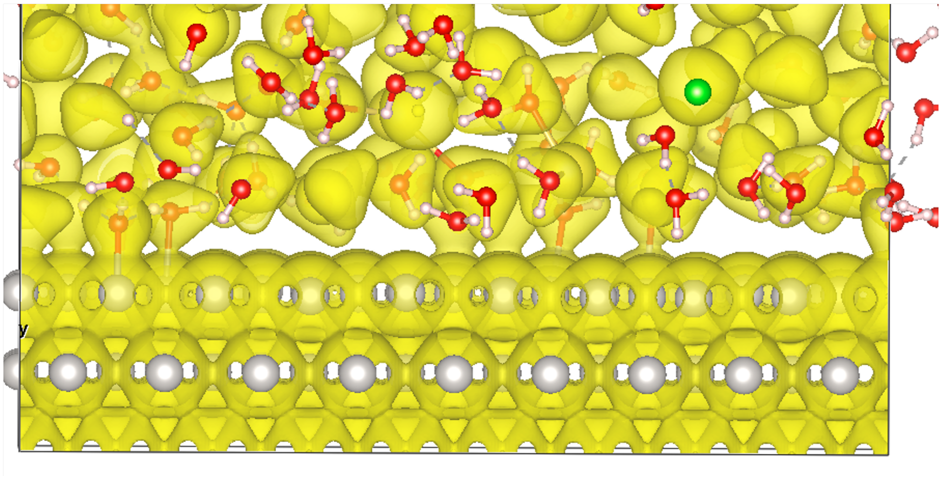

Figure S9: **Prototypic electronic charge density distribution** Electronic charge density distribution on a sample snapshot, highlighting the presence of charge along the Pt-water bond.

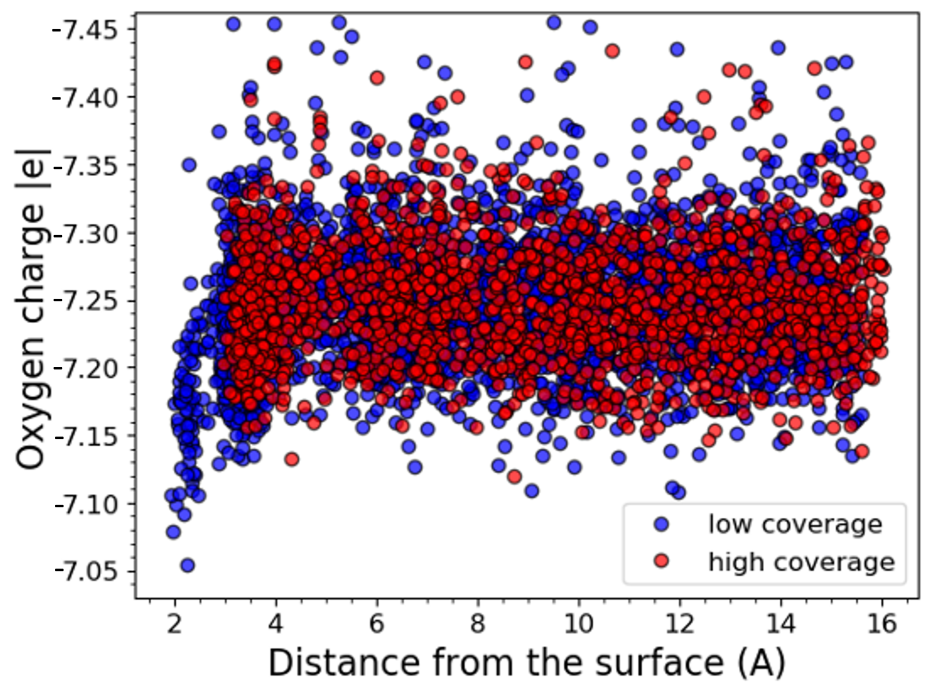

Figure S10: **Average electron charge around O atoms in units of $|e|$.** At low H coverage, the O atoms approach the metal surface more closely than at high H coverage, and they carry about 0.1–0.2 —e— more positive charge than the bulk water average.

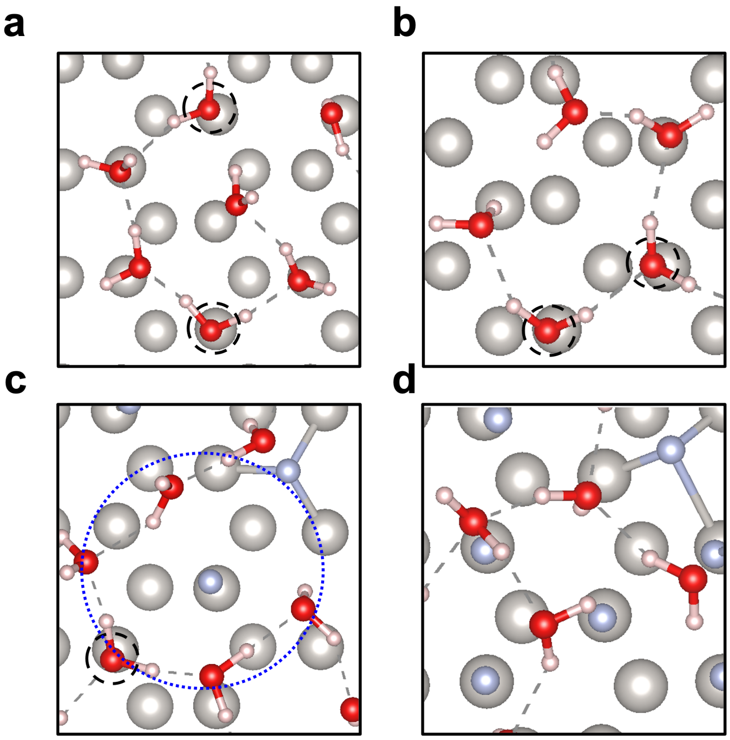

Figure S11: **Water arrangement around adsorbed Hydrogen atoms.** Water arrangement on the bare surface (a and b), next to an adsorbed hydrogen in top position (c) and on a completely covered surface (d). The atomic color follow the scheme illustrated in Figure 1a in the main text, where the hydrogen atoms adsorbed on the surface are recolored in light blue. The dashed black circles indicate an oxygen atom belonging to the first water layer. The blue circle is a guide for the eye for the arrangement of water molecules around an adsorbed top hydrogen.

Table 1: Adsorbed Hydrogen charge. Net charge of the adsorbed H_{OPD} and underlying Pt atoms on the surface, depending on their proximity with water molecules at high coverage.

	Next to water oxygen	Next to water hydrogen	Away from water
H	+0.06 $\pm$ 0.02	-0.05 $\pm$ 0.02	0.00 $\pm$ 0.01
Pt	-0.03 $\pm$ 0.01	-0.03 $\pm$ 0.01	-0.01 $\pm$ 0.02

Supplementary discussion 3: Thermodynamic origin of cluster stabilisation

To understand the thermodynamic origin of the cluster stabilisation, we compared different structures both in vacuum and in water. As a first test, the Pt electrode was covered with hydrogen atoms up to 0.33 ML in vacuum. In one case, the adsorbed atoms were equally spaced, in the other, they all formed a single cluster. Results indicate a similar stabilisation in both cases. The adsorption energy of the latter structure is -0.031 eV/Å^2, while for the former it is -0.028 eV/Å^2. Therefore, adsorbate-adsorbate interaction is not the reason for clusterisation. Water molecules must play an important role. To prove it we selected a few snapshots from the dynamics. In particular two cases where selected: a snapshot of the early stage of the dynamics when hydrogen did not cluster yet but the water had the time to properly arrange at the surface, and a snapshot of the later stage where one cluster formed surrounded by a few isolated hydrogen atoms. The energy of the two structures was calculated without any water on top of the contact and with just the first water layers. When no water was adsorbed the two structures shared the same formation energy: -0.025 eV/Å^2. With the first water layers, the structure with one cluster has an adsorption energy of -0.024 eV/Å^2 compared to the -0.021 eV/Å^2 of the one with only isolated hydrogen atoms. The H_2O arrangement is less disrupted by the presence of two distinct surfaces (bare and fully covered) rather than the presence of many isolated hydrogen atoms. In Figure **??**, a few examples of how the water arranges next to the surface are shown. It is possible to see that, when the surface is free from adsorbed hydrogen atoms, water organizes in a loosely bound geometrical arrangement which can freely change over time (Figure **??**a-b). If a hydrogen occupies the top site (one of the most common site when hydrogen atoms are isolated at low coverage), the water ordering is forced into a wider circle (dashed blue line in Figure **??**c) and can no longer freely arrange, as also discussed using an electronic structure argument in reference (***?***). The freedom in arrangement is recovered once the surface is completely covered, although there is a trend to create more packed structures (Figure **??**d). The minor steric repulsion of the clustered surface has less impact on the water compared to the other surface. As a result, in the former arrangement the number of hydrogen bonds is maximised.

The analysis of the charge of the hydrogen adsorbates provides an electronic structure-based justification for the formation of hydrogen clusters and a microscopic interpretation of the complex interactions between platinum, hydrogen, and water. Specifically, the excess Bader charges for hydrogen adsorbed in an fcc position (H_{OPD}) are consistently slightly negative, ranging between -0.10 and -0.07. This indicates that hydrogen adsorbed over the surface remains in a neutral oxidation state and does not interact significantly with water. The charges for hydrogen adsorbed on top sites

(H_{OPD}) range between -0.07 and 0.08 (see Supplementary Table **??**). This oscillation between positive and negative charges correlates with the circular configuration of the water layer above these hydrogen atoms (see Figure **??**) and generates a lateral modulation of the surface charge possibly anticipating HER.
Specifically, adsorbed hydrogen atoms remain neutral if no water is directly above them, and their charges oscillate between slightly positive and negative values depending on the orientation of the water molecules. This alternating charge scenario, supported by electronic structure calculations, explains the observed formation of hydrogen clusters (Figure 1 in main text) and provides microscopic interpretation of various literature describing with continuum models the complexity of the behaviour of adsorbates on metal substrates (*?, ?, ?, ?, ?*).

Supplementary References

1. R. Khatib, A. Kumar, S. Sanvito, M. Sulpizi, C. S. Cucinotta, The nanoscale structure of the pt-water double layer under bias revealed. *Electrochimica Acta* **391**, 138875 (2021).

2. S. Surendralal, M. Todorova, J. Neugebauer, Impact of water coadsorption on the electrode potential of h-pt(1 1 1)-liquid water interfaces. *Phys. Rev. Lett.* **126**, 166802 (2021).

3. V. Kratsov, A. Mal'Shukov, Structural and electronic alterations in an adsorbed layer. *Journal of Experimental and Theoretical Physics - J. Exp. Theor Phys.* **48** (1978).

4. S. Gudmundsdóttir, E. Skúlason, K.-J. Weststrate, L. Juurlink, H. Jónsson, Hydrogen adsorption and desorption at the pt(110)-(1×2) surface: experimental and theoretical study. *Phys. Chem. Chem. Phys.* **15**, 6323-6332 (2013).

5. K. Christmann, G. Ertl, T. Pignet, Adsorption of hydrogen on a pt(111) surface. *Surface Science* **54**, 365-392 (1976).

6. A. A. Kornyshev, W. Schmickler, On the coverage dependence of the partial charge transfer coefficient. *Journal of Electroanalytical Chemistry and Interfacial Electrochemistry* **202**, 1-21 (1986).

7. A. A. Koverga, E. Flórez, C. Jimenez-Orozco, J. A. Rodriguez, Not all platinum surfaces are the same: Effect of the support on fundamental properties of platinum adlayer and its implications for the activity toward hydrogen evolution reaction. *Electrochimica Acta* **368**, 137598 (2021).
