# Supplementary material for: Revealing the molecular interplay of coverage, wettability, and capacitive response at the Pt(111)-water solution interface under bias
Source: Commun Chem. 2025 Feb 24;8:58. doi: 10.1038/s42004-025-01446-w (PMC11850831; doi:10.1038/s42004-025-01446-w)
Supplement: Supplementary file 3 — Description of Additional Supplementary Files [file 42004_2025_1446_MOESM3_ESM.pdf]

Description of Additional Supplementary Files

File name: Supplementary Data 1 (Supplementary_data_1_0ML_i.xyz)

Description: Initial configuration in xyz format for the system with no hydrogen coverage

File name: Supplementary Data 2 (Supplementary_data_2_0ML_f.xyz)

Description: Final configuration in xyz format for the system with no hydrogen coverage

File name: Supplementary Data 3 (Supplementary_data_3_1ML_i.xyz)

Description: Initial configuration in xyz format for the system with 1ML hydrogen coverage

File name: Supplementary Data 4 (Supplementary_data_4_1ML_f.xyz)

Description: Final configuration in xyz format for the system with 1ML hydrogen coverage

File name: Supplementary Data 5 (Supplementary_data_5_033ML_i.xyz)

Description: Initial configuration in xyz format for the system with 0.33ML hydrogen coverage

File name: Supplementary Data 6 (Supplementary_data_6_033ML_f.xyz)

Description: Final configuration in xyz format for the system with 0.33ML hydrogen coverage

File name: Supplementary Data 7 (Supplementary_data_ 7_067ML_i.xyz)

Description: Initial configuration in xyz format for the system with 0.67ML hydrogen coverage

File name: Supplementary Data 8 (Supplementary_data_8_067ML_f.xyz)

Description: Final configuration in xyz format for the system with 0.67ML hydrogen coverage
